# Structural Adhesives Tapes Based on a Solid Epoxy Resin and Multifunctional Acrylic Telomers

**DOI:** 10.3390/polym13203561

**Published:** 2021-10-15

**Authors:** Mateusz Weisbrodt, Agnieszka Kowalczyk, Krzysztof Kowalczyk

**Affiliations:** Department of Chemical Organic Technology and Polymer Materials, Faculty of Chemical Technology and Engineering, West Pomeranian University of Technology in Szczecin, 70-322 Szczecin, Poland; mweisbrodt@zut.edu.pl (M.W.); kkowalczyk@zut.edu.pl (K.K.)

**Keywords:** epoxy resins, thermoset polymers, structural adhesives, acrylic monomers, telomerization, mechanical properties

## Abstract

Thermally curable pressure-sensitive structural adhesives tapes (SATs) were compounded using a solid epoxy resin and multifunctional acrylic telomer solutions (MATs) prepared by a thermally initiated telomerization process in an epoxy diluent containing two kinds of telogens (CBr_4_ or CBrCl_3_). Dynamic viscosity, K-value, and volatile mater content in MATs (i.e., MAT-T with CBr_4_, MAT-B with CBrCl_3_) were investigated in relation to telogen type and content. The influence of MATs on the self-adhesive features and curing behavior of UV-crosslinked tapes as well as on the shear strength of thermally cured aluminum–SAT–aluminum joints was investigated as well. Increasing the telogen dose (from 5 to 15 wt. parts) caused significant improvement in the adhesion (+315% and +184%), tack (+147% and +298%), and cohesion (+414% and +1716%) of SATs based on MAT-T and MAT-B, respectively. Additionally, MATs with high telogen content (especially the MAT-T-type) improved the resistance of cured joints to aviation fuel, humidity, and elevated temperature. The highest overlap shear strength values were registered for SATs based on MATs containing 7.5 wt. parts of CBr_4_ (16.7 MPa) or 10 wt. parts of CBrCl_3_ (15.3 MPa).

## 1. Introduction

The field of application of epoxy resins (ERs) as structural binders and polymer matrices is constantly expanding [1,2,3,4]. Adhesives based on ERs are classified as reactive systems and show excellent properties, especially when joining ceramics and metals [5,6,7]. For more than 20 years, epoxy adhesives have been used in the aerospace, automotive, and marine industries as well as other special industrial applications [8,9]. The importance of adhesives in these areas has significantly increased with the increasing utilization of modern composite materials and light alloys (e.g., aluminum-based alloys) [10]. Generally, the highest shear resistance bonds are formed using structural adhesives. These adhesives are typically applied as a paste or a film/sheet. The resulting structural adhesive films (SAFs) consist of a thin precatalyzed resin layers supported on a scrim or paper backing; various types of scrims enhance handling of the adhesive and allow control over the thickness of the created bonds [11,12]. Nevertheless, the application of SAFs is not very convenient, as can be observed in the case of structural adhesive tapes (SATs), i.e., unsupported thermosetting tapes exhibiting self-adhesive properties. The latter systems have been claimed to be a new generation of structural adhesive materials [13,14,15].

Cured epoxy resins are very important adhesive materials because of their relatively high mechanical strength, stiffness, and high-temperature resistance. However, their low-temperature and thermal shock resistance, vibration resistance, adhesion, and shear strength need to be enhanced. To develop high-performance adhesives, ERs have been modified by incorporation of additives such as reactive diluents, fillers, and flexibilizers [16,17,18,19,20,21]. In order to increase the self-adhesive properties of epoxy-based structural adhesives, various acrylic copolymers have been tested [22]; it was revealed that polymeric derivatives of butyl acrylate and glicydyl methacrylate effectively improved the impact strength of ERs as well [23]. It should be noted that the efficacy of modifications for a given adhesive (and its compatibility with ERs) strongly depends on its molecular weight.

Generally, multifunctional telomers are prepared via telomerization of a functional monomer(s). Telomerization is a reaction between an XY-type compound (the *telogen*) and a polymerizable M molecule called the *taxogen* (i.e., the monomer(s)). The process can be initiated by radical initiators or redox catalysts [24,25] and—the most importantly—it results in the X(M)_n_Y polymers with low molecular weights (oligomers/telomers). Acrylic monomers (mainly with carboxyl, hydroxyl, epoxy, amine, or amide-type functional groups) are commonly used for the preparation of multifunctional telomers. Numerous chemical transformations of polyacrylic telomers have been carried out in order to fulfill interesting industrial applications such as antifouling paint binders [26,27,28,29].

This contribution presents a preparation method for multifunctional acrylate telomers (MATs) containing hydroxyl, epoxy, and photoreactive benzophenone groups that are based on two telogens (i.e., terabromomethane (CBr_4_) or bromotrichloromethane (CBrCl_3_)). The prepared MATs, with terminal halogen groups -CBr_3_ or -CCl_3_, were applied as modifiers of a solid epoxy resin in order to obtain thermosetting structural tapes with self-adhesive properties (SATs). Generally, the influence of telogen type and concentration on the thermally initiated telomerization process of selected (meth)acrylate monomers (in the presence of epoxy diluent) was investigated. Additionally, the (thermo)mechanical features of structural adhesives containing the prepared telomers were tested and are discussed in detail.

## 2. Materials and Methods

### 2.1. Materials

The following compounds were used for the preparation of the multifunctional acrylate telomers (MATs): n-butyl acrylate (BA; Merck, Germany), glycidyl methacrylate (GMA; Dow Europe, Switzerland), 2-hydroxyethyl acrylate (HEA; Poly-Chem, Germany), 4-acryloyloxybenzophenone (ABP; Chemitec, Germany), 2,2′-azobis(isobutyronitrile) (AIBN; Merck, Germany), trimethylolpropane triglycidyl ether (Grilonit V51-31; EMS-CHEMIE, Switzerland), and tetrabromomethane (TBM) and bromotrichloromethane (BTChM) (Sigma-Aldrich, St. Louis, MO, USA).

The Bisphenol A-based solid epoxy resin (epoxy equivalent weight of 370 g/equiv., Epidian 2; Ciech Sarzyna, Poland), the micronized dicyandiamide-based curing agent (Epikure P-104; Hexion, Oklahoma, OK, USA), pentaerythritol triacrylate (PETIA, Allnex, Bergen, The Netherlands), the radical photoinitiator (Omnirad 127; IGM Resins, Waalwijk, The Netherlands), and the surface tension-reducing compound (Byk 4510; Altana AG, Wesel, Germany) were used as components of the self-adhesive structural tapes (SATs).

### 2.2. Preparation and Characterization of the Multifunctional Acrylic Telomers (MATs)

MATs were prepared via bath telomerization of BA, GMA, HEA, and ABP (used as three monomers and a polymerizable photoinitiator, respectively) in the presence of the telogens (TBM or BTChM) and the epoxy diluent (V51-31). The process was thermally initiated by AIBN at 90 °C in a glass reactor equipped with an inert gas supply, a mechanical stirrer, and a thermocouple. The reaction mixture consisted of 100 wt. parts of the monomers, 1.2 wt. parts of AIBN, and 5, 7.5, 10, or 15 wt. parts of the telogen. The reaction mixture was added dropwise for 90 min to the heated epoxy diluent (20 wt. parts); then, the telomerization process was continued for 3 h. The composition of the MATs is specified in Table 1.

### 2.3. Preparation of the Self-Adhesive Tapes (SATs) and Al/SAT/Al Joints

SATs were compounded using MAT (50 wt. parts), Epidian 2 (50 wt. parts), Epikure P-104 (2 wt. parts) and Byk 4510 (0.5 wt. part). Moreover, PETIA (5 wt. parts) and Omnirad 127 (2.5 wt. parts) were added in order to increase the cohesion of SATs after UV crosslinking. Generally, the aforementioned components (after dissolution of the epoxy resin in the system) were homogenized at 60 °C for 15 min (500 rpm) by the RE16 mechanical mixer (IKA, Germany). Then, the composition was applied onto a polyester foil (samples for self-adhesive tests) or a siliconized paper (other tests) and UV-irradiated for 48 s (12 J/cm^2^) using the UV-ABC medium-pressure mercury lamp (Hönle UV-Technology, Gräftingen, Germany). The UV exposition was controlled by the Dynachem 500 radiometer (Dynachem, Westville, IL, USA). The thickness of the UV-crosslinked SAT layers was 200 µm. Aluminum–SAT–aluminum overlap joints (Al/SAT/Al) were prepared using the SATs and degreased 2024 aluminum panels (100 × 25 × 1.6 mm). The joints were thermally cured at 170 °C for 60 min.

### 2.4. Methods

#### 2.4.1. Characterization of MATs

##### Dynamic Viscosity

The dynamic viscosity of the MAT solutions was determined at room temperature using the DV-II Pro Extra viscometer (spindle #7, 50 rpm; Brookfield, New York, NY, USA).

##### Volatile Matter Content

The volatile matter content in the MAT solutions was determined by a thermogravimetric method; an MAT solution sample was heated at 105 °C for 4 h in a moisture analyzer (MA 50/1.X2.IC.A; Radwag, Radom, Poland).

##### Glass Transition Temperature

Glass transition temperature (T_g_) values of the MAT solutions were determined using the Q100 differential scanning calorimeter (TA Instruments, New Castle, DE, USA). Samples (ca. 10 mg) were analyzed using hermetic aluminum pans at temperatures from −80 to 380 °C (heating rate of 10 °C/min).

##### K-Value

The K-values for MATs were determined using an Ubbelohde viscometer according to the EN ISO 1628-1:1998 standard and the Fikentscher Equation (1) [30]:

(1) $$k=\frac{1.5\mathrm{lg}\;\eta_{r}-1+\sqrt{1+\left(\frac{2}{c}+2+1.5\mathrm{lg}\eta_{r}\right)1.5\mathrm{lg}\eta_{r}}}{150+300c}$$

where: *η_r_* = *η/η*_0_; *η*—viscosity of a polymer solution (i.e., the MAT solutions); *η*_0_—viscosity of a pure auxiliary diluent (i.e., tetrahydrofurane); *c*—polymer concentration (g/cm^3^).

#### 2.4.2. Characterization of Thermally Uncured SATs

##### Self-Adhesive Properties

Adhesion of UV-crosslinked (thermally uncured) SATs to steel substrate was determined at the angle of 180° according to the AFERA 5001 standard developed by the European Association for Testing Adhesives for the Automotive Industry (AFERA) using a Z010 testing machine (Zwick/Roell, Ulm, Germany). SAT samples (175 × 25 mm) were applied onto a degreased steel plate and pressed with a rubber roller (2 kg) in order to improve the wettability of the substrate by the tested adhesive. The adhesion test was performed 20 min after film application with a peeling speed of 300 mm/min. Five measurements of each SAT were performed.

Tack was determined by the loop method in accordance with the AFERA 5015 standard using the Z010 testing machine. An SAT film (175 × 25 mm) was mounted in the upper jaw of the machine to obtain a loop with the adhesive layer on the outside. The sample was perpendicularly lowered (100 mm/min) onto a degreased steel plate placed in the lower jaw. The contact area was ca. 6.25 cm^2^. The machine recorded the force value needed to detach the adhesive film after a short contact (ca. 0.5 s) with the steel surface and without external forces. The result was the average value of five consecutive measurements.

Cohesion (i.e., static shear adhesion) describes the time needed to shear off an adhesive tape sample (under a load of 1 kg) from a defined steel surface. Cohesion was determined in accordance with the AFERA 5012 standard using a device designed by the International Laboratory of Adhesives and Self-Adhesive Materials (WPUT in Szczecin), which enables automatic measurement of the time of joint crack occurrence.

##### Characterization of the SAT Thermal Curing Process

Differential scanning calorimetry (DSC Q100, TA Instruments, New Castle, DE, USA) was used for determination of the onset temperature of the curing reaction (T_i_), the maximum temperature of the curing reaction (T_p_), and the enthalpy of the SAT curing process (ΔH). Hermetic aluminum DSC pans were used, and samples (ca. 10 mg) were analyzed from −80 to 350 °C (heating rate of 10 °C/min). Additionally, the T_g_ values for thermally uncured SATs were determined. Two DSC measurements for each system were carried out.

#### 2.4.3. Characterization of Thermally Cured SATs and Al/SAT/Al Joints

##### Overlap Shear Strength

The overlap shear strength of the Al/SAT/Al systems was measured at room temperature according to the ASTM D1002-10 standard (ten samples of each system) using the Z010 machine (Zwick/Roell, Ulm, Germany). The humidity resistance of the joints was determined according to the MMA-A-132B standard after their 30-day exposure (40 °C, 95% RH) in an LHU-114 climatic chamber (Escpec, Osaka, Japan). A fluid immersion test was performed (MMM-A-132B) after immersion of the Al/SAT/Al joints at 23 °C for 7 days in aviation turbine fuel (Jet A-1; PKN Orlen, Warszawa, Poland). A long-lasting thermal ageing test (MMM-A-132B) was conducted as well; the shear strength of the joints was measured after their storage at 82 °C for 192 h.

##### Crosslinking Degree

The crosslinking degree (α) of the thermally cured SATs was calculated using DSC data according to Equation (2) [31].

(2) $$\alpha =\left(\frac{\Delta H_{T-}\Delta H_{res}}{\Delta H_{T}}\right)\;\left(a.u.\right)$$

where: $\Delta H_{T}$—total enthalpy of the SAT curing process (J/g); $\Delta H_{T}$—enthalpy of the postcuring process of the thermally cured SAT (in an Al/SAT/Al joint).

##### Thermomechanical Properties

The thermally cured SATs were tested for their thermomechanical properties using the DMTA Q800 analyzer (TA Instruments, New Castle, DE, USA). The SAT sheets were cut into coupons with dimensions of ca. 50 × 10 mm; the final samples (total thickness of 2 mm) were prepared via a coupon-by-coupon assembly method. The test was performed using a dual cantilever (a flexural mode) at temperatures from −80 to 160 °C (heating rate of 2 °C/min). The deformation frequency was 1 Hz.

## 3. Results

### 3.1. Properties of MATs

**Figure 1:**
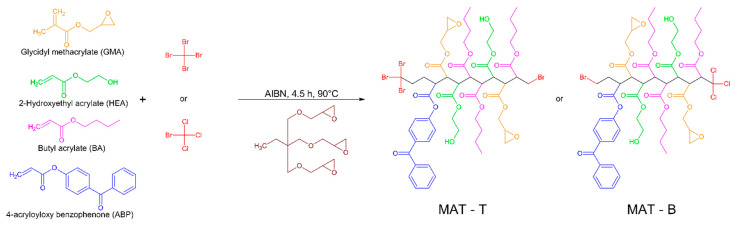
Theoretical chemical structures of the monomers, telogens, and acrylic telomers (MAT-T with tetrabromomethane; MAT-B with bromotrichloromethane).

Two groups of MATs with different telogens, i.e., tetrabromomethane (TBM) and bromotrichloromethane (BTChM), were obtained via a thermally initiated telomerization process. The products (the MAT solutions in the epoxy diluent, 80/20 m/m) contained built-in epoxy (from GMA), hydroxyl (from HEA), and photoreactive benzophenone groups (from ABP) as well as the terminal halogen atoms Br (MAT-T with TBM) or Br and Cl (MAT-B with BTChM); the theoretical structures of the MATs are presented in Figure 1. Values of the dynamic viscosity and volatile mater content for the MAT solutions are presented in Table 2.

**Figure 2:**
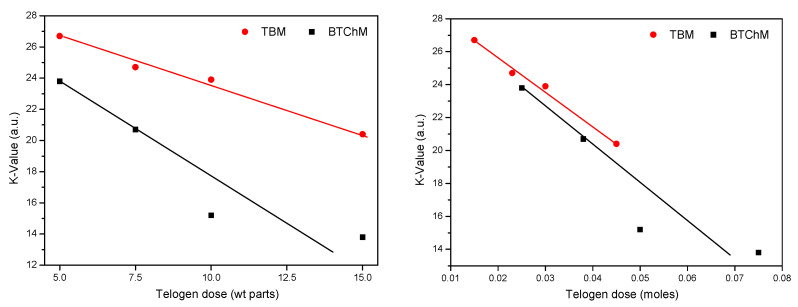
K-values of multifunctional acrylic telomer solutions in relation to the type and dose (by weight or moles) of the telogens.

The viscosity values for the MAT-T solutions were ca. two times higher than those for the MAT-B type; only the systems with the highest telogen content (15 wt. parts/100 wt. parts of the monomers) reached similar values for this parameter (3.5 Pa·s and 3.6 Pa·s). Generally, the results showed the important relation between viscosity and telogen content (regardless of its type): the higher the telogen concentration, the lower the viscosity of the MAT solutions. On the other hand, the type of telogen affected the volatile matter content in the systems. In the case of the MATs-T, volatile matter content increased from 2.8 wt% (MAT-T-5) to 4.9 wt% (MAT-T-15). This may have been caused by the presence of unreacted monomers and/or the formation of a series of extraordinary low-molecular-weight volatile products in the TBM-based systems. Interestingly, the volatile compound content in the MAT-B compositions was relatively low and unaffected by the BTChM content (3.3 wt% for MAT-B-5 and 3.1 for MAT-B-15). Probably, the aforementioned relatively high volatile component content in MAT-T-15 caused significant reduction in its viscosity in relation to MAT-T-10 (4.9 wt% and 3.6 Pas vs. 3.7 wt% and 15.4 Pas, respectively). The same phenomenon was not observed for MAT-B-15 (3.1 wt%, 3.5 Pa·s) and MAT-B-10 (3.1 wt%, 7.8 Pa·s). Nevertheless, the higher viscosity of the MAT-T samples (in relation to the MAT-B systems with similar solid content) may indirectly indicate their higher average molecular weights. This was confirmed by the results of the K-value test, which expresses the molecular weight of polymeric materials; the relation between the K-value and the type (and content) of telogen is presented in Figure 2. Generally, higher K-values were registered for the samples with TBM (27 a.u. for MAT-T-5 and 20 a.u. for MAT-T-15) than for the BTChM-based compositions (24 a.u. for MAT-B-5 and 14 a.u. for MAT-B-15); this indicates that the MAT-T systems were characterized by a higher molecular weight. Considering the telomerization mechanism, it can be claimed that the observed differences in the K-values partly resulted from the varying molecular weights of the applied telogens (331.6 g/mol for TBM, 198.3 g/mol for BTChM). Thus, the related MAT-T and MAT-B systems contained different numbers of telogen molecules; for any given pair of samples (e.g., MAT-T-5 and MAT-B-5), a lower telogen molecule concentration was noted in the TBM-based systems (0.015 mole of TBM or 0.025 mole of BTChM per 100 wt. parts of the monomers mixture). Nevertheless, the MATs-B reached slightly lower K-values (i.e., molecular weights) than the corresponding MAT-T samples (Figure 2).

**Figure 3:**
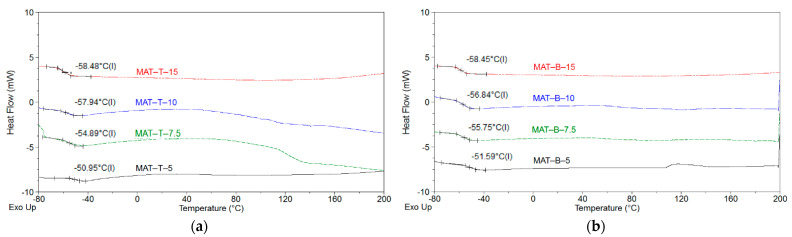
DSC thermographs for MAT-T (**a**) and MAT-B solutions (**b**).

Since the MAT solutions would be used as a main component of the pressure-sensitive structural adhesive tapes (SATs), their glass transition temperature (T_g_) values were examined; the DSC test results are shown in Figure 3. It is generally known that the T_g_ of typical polyacrylate pressure-sensitive adhesives (PSAs) is lower than −20 °C. Above this temperature, the self-adhesive features of PSAs are unsatisfactory. Notably, DSC thermographs revealed only one T_g_ value for each sample. This means that all the MAT solutions contained statistical co-oligomers (cotelomers). On the other hand, both types of MAT systems (with TBM or BTChM) were characterized by very low and similar T_g_ values (ca. −51 °C for MATs-5 and −58 °C for MATs-15). The lower T_g_ values registered for the samples with the higher telogen content resulted from the lower molecular weights of the copolymers/co-oligomers, because smaller molecules generally exhibit higher mobility. Thus, it can be expected that the telomers (as parts of polymeric networks) positively affect the self-adhesive properties (adhesion, tack, cohesion) of UV-crosslinked SATs.

### 3.2. Self-Adhesive Features of SATs Based on the MAT Solutions

**Figure 4:**
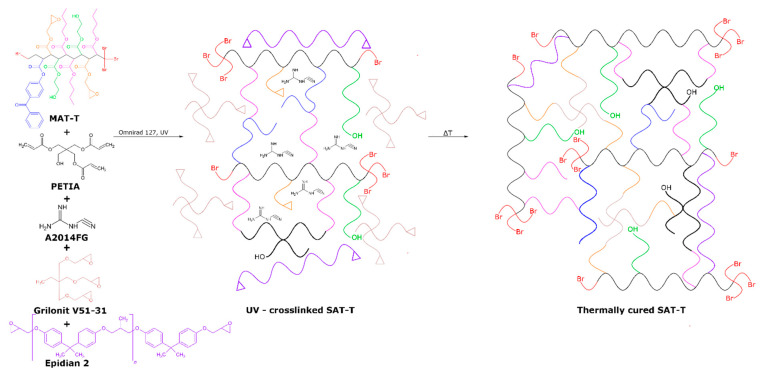
Preparation steps of UV-crosslinked SATs and thermally cured SATs.

**Figure 5:**
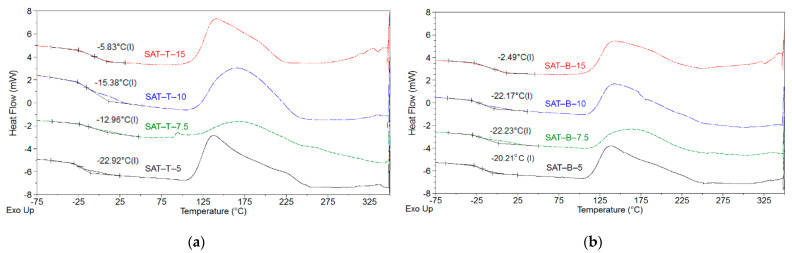
DSC thermographs for UV-crosslinked SATs based on MAT-T (**a**) or MAT-B (**b**).

The MAT-T and MAT-B solutions (in the epoxy diluent), as well as the solid epoxy resin, the thermally activated latent curing agent, pentaerythritol triacrylate, and the UV-photoinitiator, were used for preparation of the structural adhesive compositions. After the UV irradiation of the aforementioned components (in thin film form), thermally curable pressure-sensitive structural adhesive tapes (SATs) were obtained (Figure 4). In this study, the basic self-adhesive features (i.e., adhesion, tack and cohesion) of MAT-T- and MAT-B-based SATs were investigated. As previously mentioned, the self-adhesive properties of SATs (as in the case of typical pressure-sensitive adhesives) depend—among other factors—on their glass transition temperature, which should be lower than −20 °C. DSC thermographs and T_g_ values for the prepared SATs (after the UV crosslinking but before the thermal curing process) are shown in Figure 5.

As can be seen, only the SATs-B with low-to-medium BTChM content (5, 7.5, or 10 wt. parts) and SAT-T-5 (5 wt. parts of TBM) exhibited acceptably low T_g_ values (from −23 to −20 °C). Generally, the higher the telogen concentration in SATs, the higher the T_g_ values of the system were (this relation was especially observed for SATs-T). This shows that SATs based on MATs with relatively lower molecular weights (i.e., with lower K-values) exhibited higher T_g_ values. This phenomenon is opposite to that observed for the reference MAT solutions (the T_g_ decreased with increasing telogen content and decreasing molecular weight value). The key difference is that the SATs were compounded using solid epoxy resin (ER) and a triacrylate component (PETIA), and they were UV crosslinked. The latter process caused the formation of a semi-interpenetrating polymer network (consisting of the thermoplastic ER and a MAT/PETIA-based 3D-type web structure) (Figure 4). Arguably, the UV crosslinking of the adhesive compositions containing MATs (with the relatively lower viscosity and molecular weight values) was more effective than that of the other systems. Thus, more dense acrylate polymer networks were formed; this resulted in lower mobility of linear parts of the networks and high T_g_ values.

DSC analyses of the thermally initiated curing process of UV-crosslinked SATs revealed that the tapes with the relatively denser polyacrylate networks (i.e., the systems with the T_g_ values higher than −20 °C: SAT-T-7.5, SAT-T-10, SAT-T-15, and SAT-B-15) were characterized by higher onset temperature (T_i_), higher maximum temperature (T_p_), and lower enthalpy of the curing process (ΔH, Table 3) than the other samples. Generally, the specific thermal parameters values significantly depended on the MAT type. Additionally, changes of the most important feature, ΔH, probably resulted from different epoxy diluents and epoxy-functional telomer weight contents in the MATs used for SAT preparation (due to varied telogens doses from 5 to 15 wt. parts per constant mixture weight of the other components).Thus, SATs-7.5, SATs-10, and SATs-15 contained 1.9, 3.8, and 7.3 wt% less of the aforementioned epoxy components than the reference systems (i.e., SAT-B-5 and SAT-T-5).

**Figure 6:**
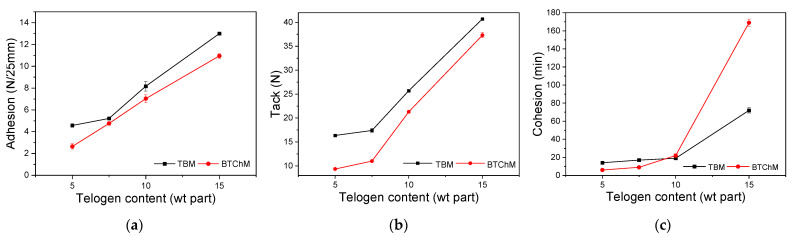
Adhesion to steel (**a**), tack (**b**), and cohesion (**c**) of SATs in relation to the type and content of their telogens.

As mentioned above, the self-adhesive features of the UV-crosslinked SAT samples were tested before their thermal curing; results are shown in Figure 6. Adhesion to steel substrate was improved from 4.5 to 13 N/25 mm (SAT-T-5, SAT-T-15) and from 3 to 11 N/25 mm (SAT-B-5, SAT-B-15). Tack changed from 16.3 to 40.7 N and from 9.3 to 37.3 N, respectively. Interestingly, values of these parameters increased with increasing telogen content, however, the latter parameter increment (i.e. telogen content not tack) caused an increment of Tg (even above −20 °C) and crosslinking density of the SATs. This direct relation is not typical for PSA-type products.

The test results showed that adhesion to steel substrate and tack significantly depended on the type and number of terminal groups in the polyacrylate chains, which arguably interacted with the polar surface of the stainless steel. The adhesion improvement can be partly explained by the electrostatic theory of adhesion.

The polyacrylate networks in the SATs—thanks to the use of appropriate telogens (CBr_4_ or CBrCl_3_)—contained characteristic terminal groups (-C-Br_3_ in SAT-T or -C-Cl_3_ in SAT-B) instead of -C-H_3_. It is known that electronegativity of bromine (2.8) and chlorine (3.0) is higher than that of hydrogen (2.1) (on the Pauling scale) [32]. According to the electrostatic adhesion theory, higher electronegativity means a larger flow of electrons between adhered materials [33]. Thus, the presence of more electronegative groups in the SATs (more terminal halogen groups) should cause higher adhesion and tack. This theory explains the positive influence of halogen concentration on adhesion and tack, but it does not explain the higher adhesion and tack of the SAT-T samples (Br atoms) in relation to the SAT-B samples (Cl atoms). Perhaps it resulted from the lower T_g_ values of the former systems (e.g., −5.8 °C for SAT-T-15 and −2.5 °C for SAT-B-15).

The highest cohesion values (ca. 170 and 70 h) were registered for the SAT-B-15 and SAT-T-15 samples (Figure 6c); these samples were characterized by the lowest K-values of introduced MATs (ca. 14 a.u. and 20 a.u., respectively) and the highest T_g_ after the UV crosslinking process in relation to the other systems. It seems that the crosslinking density of the SATs was a crucial parameter affecting cohesion.

#### Properties of Al/SAT/Al Joints

**Figure 7:**
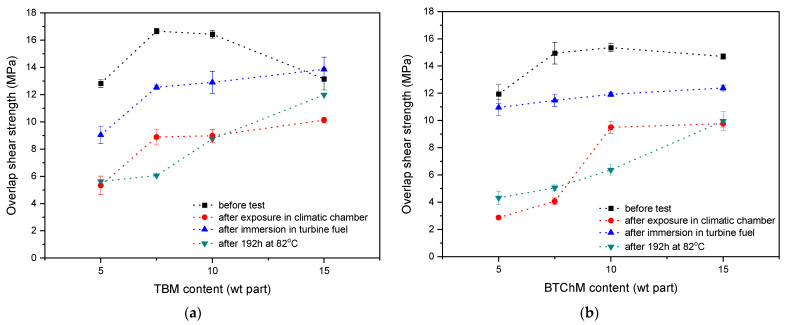
Overlap shear strength (before and after aging tests) of Al/SAT/Al joints based on MAT-T (**a**) and MAT-B (**b**).

The UV-crosslinked SATs were applied between aluminum panels and thermally cured at 170 °C for 60 min. Additionally, the prepared Al/SAT/Al joints were thermally aged, immersed in turbine fuel, or exposed in a climatic chamber. Overlap shear strength (τ) values for the joints are presented in Figure 7. Generally, τ of the initial joints (i.e., after thermally curing, no aging) was higher than the value required for structural adhesives according to the ASTM standard (7 MPa).

In detail, higher telogen concentrations (up to 10 wt. parts) increased the τ values: from 12.8 MPa (SAT-T-5) to 16.4 MPa (SAT-T-10) and from 11.9 MPa (SAT-B-5) to 15.3 MPa (SAT-B-10). Lower results were noted for the systems based on MAT-B. As mentioned above, the SAT-B samples exhibited overall denser polyacrylate networks—formed during the UV-crosslinking stage—because MAT-B was distinguished by a lower molecular weight (the lower K-values) and viscosity. It is known that the shear strength of structural adhesives depends on their crosslinking degree, and an optimal range of the latter parameter can be often observed [34]. The α values of the SATs (Table 3) were higher than 0.9 a.u. The full crosslinking degree was calculated for SAT-T-15 and the SAT-B-type systems (except for SAT-B-15; 0.95 a.u.). The noted reduction of the α parameter (SAT-B-15 vs. the other SAT-B samples) was probably caused by the relatively low molecular weight (the K-value) of MAT-B-15; cationic polymerization of epoxy groups (from epoxy resin, epoxy diluent, and an MAT component) is generally hampered in SATs containing a dense polyacrylate network formed during a UV-crosslinking process. This was confirmed by the increment in T_i_ (from 113 to 118 °C) and reduction in enthalpy (from 146 to 123 J/g; Table 3) noted for SAT-B (and SAT-T) with the highest telogen content. Nevertheless, very high α values were achieved for all the SATs, which did not correlate with the registered high shear strength. As presented in [35], a too high α value may deteriorate the shear strength of Al/SAT/Al overlap joints.

For shear strength values recorded after different ageing conditions were applied to the thermally cured Al/SAT/Al joints, the best results were achieved for the SAT-T-type samples. The ageing processes reduced shear strength; however, there was a general trend of a positive influence of terminal halogen groups on τ. Moreover, it is noteworthy that SATs were more aviation fuel-resistant (−29% for SAT-T-5 and −22% for SAT-B-7.5 and SAT-B-10 vs. the relevant unaged samples) than resistant to humid conditions. On the other hand, similar or lower τ values (in relation to the samples after the climate chamber test) were noted for joints aged at an elevated temperature (82 °C) (−64% for SAT-T-5 and −66% for SAT-B-7.5 vs. the relevant unaged samples). The lowest mechanical strength was observed for SAT-B-5 and SAT-B-7.5 after their exposure in the climatic chamber (2.9 MPa and 4.0 MPa, respectively) or after their long-lasting heating (4.3 MPa and 5.0 MPa, respectively). These samples were probably affected by the low density of their MAT-B-based polyacrylate networks, which resulted in the relatively lowest adhesion, tack, and cohesion of thermally uncured SAT-B-5 and SAT-B-7.5 (in comparison with the other SATs-B and SATs-T; Figure 6). This generally shows that a higher content of halogen groups (and a lower K-value) in the tested MATs was advantageous, because the resulting Al/SAT/Al joints had lower sensitivity to different ageing conditions.

**Figure 8:**
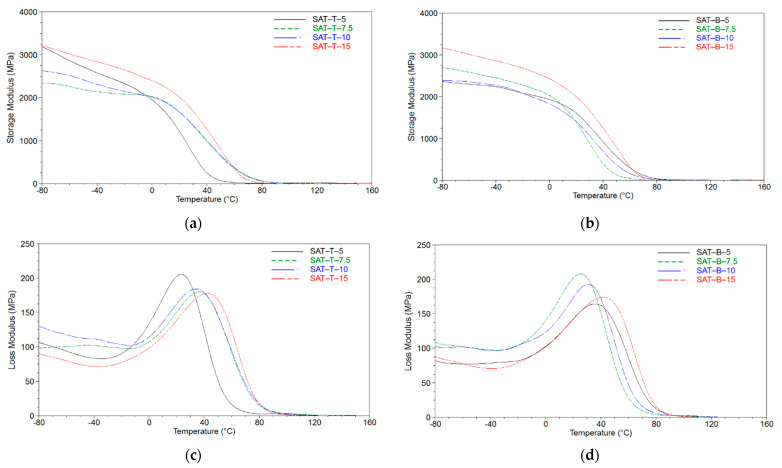

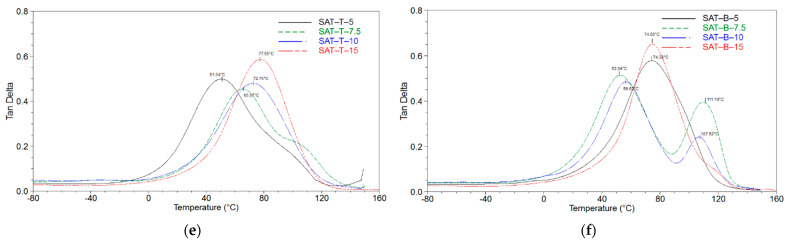
Storage module (**a**,**b**), loss module (**c**,**d**), and tan delta (**e**,**f**) for thermally cured SATs based on MATs.

Considering the thermomechanical features of the thermally cured SATs (determined by the DMTA technique; Figure 8), the storage modules for SAT-T and SAT-B were similar and reached 2300–3250 MPa at −80 °C (the highest values were registered for SATs with the highest telogen content). Nevertheless, the glassy regime was significantly wider for SATs-B than for SATs-T; the former materials were stiff up to room temperature, but their loss modulus values (at −80 °C) were slightly lower than those of the SATs-T. Recorded values of the tan delta revealed two glass transition temperatures for a few thermally cured SATs: SAT-T-5 (51 and 95 °C), SAT-T-7.5 (65 and 107 °C), SAT-B-7.5 (52 and 110 °C), SAT-B-10 (56 and 107 °C), and SAT-B-15 (74 and 120 °C). Except for SAT-B-5, the value of the main T_g_ increased (from 51 to 78 °C or from 52 to 75 °C) with increasing telogen content in the SATs-T or SATs-B, respectively; a similar relation was previously observed for thermally uncured SATs (Figure 5). Interestingly, the T_g_ values determined for the thermally uncured (−20 °C; Figure 5b) and thermally cured SAT-B-8 (74 °C; Figure 8f) were higher than those noted for SAT-B-7.5 and SAT-B-10. It is also noteworthy that only thermally cured SAT-B-5 exhibited only one T_g_ among the other SAT-B systems. Generally, the double T_g_ values might confirm the previous claim that the systems with BTChM—characterized by lower K-values (lower molecular weights) and viscosity—consisted of relatively high-density polyacrylate networks (based on MATs-B and PETIA). Probably, this resulted in phase separation of the epoxy components (the epoxy resin and diluent) and the MAT in the SATs-B during the thermal curing process. Nevertheless, these double T_g_-type materials may exhibit better impact resistance than the internally uniform materials [36].

## 4. Conclusions

In this paper, a new method of self-adhesive structural tape (SAT) preparation—using multifunctional acrylic telomers—is presented and discussed. At the beginning, the influence of different contents of the two telogens (i.e., 5, 7.5, 10, and 15 wt. parts of tetrabromomethane or bromotrichloromethane) on the thermally initiated telomerization process of (meth)acrylate monomers (containing epoxy and hydroxyl groups) in the presence of an epoxy diluent was investigated. The prepared telomers (MAT-T and MAT-B as the main components of SATs-T and SATs-B, respectively) and a solid epoxy resin were used. The physicochemical features of the MATs and their impact on the UV-crosslinking and thermal curing processes of SATs were analyzed. Generally, MATs with higher telogen content exhibited lower viscosity and K-value (molecular weight), but higher content of volatile matter (especially in the samples with CBr_4_). Thus, it can be claimed that CBrCl_3_ was the more effective telogen (a chain transfer agent). However, both types of MATs had similarly low T_g_ values (from −58 to −51 °C, depending on the telogen content). The features of the MATs influenced the UV-crosslinking process and self-adhesive properties of the thermally uncured SATs. Better properties were exhibited by SATs based on MATs-T (with CBr_4_); adhesion to steel, tack, and cohesion increased with increasing telogen content in MATs. This phenomenon was attributed to electrostatic interactions of terminal end groups of MATs (present in the UV-crosslinked SATs as a polyacrylate network) with the steel surface. Additionally, the initial temperature and enthalpy of the thermal curing process were affected by the telogen content in the applied MATs (a high density of the polyacrylate network in UV-crosslinked SATs systems disturbed the cationic polymerization of epoxy groups located in the epoxy resin, diluent, and MATs). In the case of aluminum–SAT–aluminum joints (after a thermal curing process performed on the tested SAT materials), the highest shear strength was recorded for systems containing MAT-T (7.5 wt. parts: 16.7 MPa, 10 wt. parts: 16.4 MPa) or MAT-B (10 wt. parts: 15.3 MPa). Additionally, a positive influence of telogen content on joints’ resistance to elevated temperature, humidity, and aviation fuel was observed. Thermomechanical tests revealed two T_g_ values for several cured SATs (mainly for those based on CBrCl_3_). This confirmed phase separation of polyacrylate and epoxy resin networks in SATs. The creation of the polyacrylate network was possible thanks to the formation of low-molecular weight MAT components during the telomerization process, which was confirmed in the research.

In summary, the thermally initiated telomerization process performed on the selected acrylic monomers (and in the presence of the epoxy diluent) seems to be a new and convenient method of solvent-free adhesive binder preparation. These multifunctional acrylic telomers were initially miscible with uncured solid epoxy resin and created structural adhesive tapes with good pressure-sensitive features (after a UV-crosslinking process) and mechanical strength (after an additional thermal curing process).

## Figures and Tables

**Table 1:** Composition of multifunctional acrylate telomers (MATs).

MAT Symbol	Telogen		Epoxy Diluent (wt. Parts)	Monomers (wt. Parts)				Initiator (wt. Parts)
	Symbol	wt. Parts	V51-31	BA	GMA	HEA	ABP	AIBN
MAT-T-5	TBM	5	20	79.5	8.7	10.6	1.2	1.2
MAT-T-7.5		7.5						
MAT-T-10		10						
MAT-T-15		15						
MAT-B-5	BTChM	5						
MAT-B-7.5		7.5						
MAT-B-10		10						
MAT-B-15		15						

**Table 2:** Viscosity and volatile mater content vales for the MAT solutions.

MAT Symbol	Viscosity (Pa∙s)	Volatile Matter Content (wt%)
MAT-T-5	30.8	2.8
MAT-T-7.5	23.4	3.4
MAT-T-10	15.4	3.7
MAT-T-15	3.6	4.9
MAT-B-5	16.0	3.3
MAT-B-7.5	15.6	3.0
MAT-B-10	7.8	3.1
MAT-B-15	3.5	3.1

**Table 3:** Thermal parameters of the UV-crosslinked SATs (DSC data) and the crosslinking degree (α) of the thermally cured SATs.

SAT Symbol	MAT Type	T_i_ (°C)	T_p_ (°C)	ΔH (J/g)	α (a.u.)
SAT-T-5	MAT-T-5	113	139	166	0.91
SAT-T-7.5	MAT-T-7.5	115	179	139	0.93
SAT-T-10	MAT-T-10	119	169	122	0.94
SAT-T-15	MAT-T-15	122	141	105	1.0
SAT-B-5	MAT-B-5	113	140	146	1.0
SAT-B-7.5	MAT-B-7.5	114	142	130	1.0
SAT-B-10	MAT-B-10	115	144	125	1.0
SAT-B-15	MAT-B-15	118	167	123	0.95

T_i_—the onset temperature of the curing reaction; T_p_—the maximum temperature of the curing reaction; ΔH—enthalpy of the SAT curing process; α—the crosslinking degree of the thermally cured SATs.

## Data Availability

Not applicable.
